# Small heat-shock protein HspL is induced by VirB protein(s) and promotes VirB/D4-mediated DNA transfer in *Agrobacterium tumefaciens*

**DOI:** 10.1099/mic.0.030676-0

**Published:** 2009-10

**Authors:** Yun-Long Tsai, Ming-Hsuan Wang, Chan Gao, Sonja Klüsener, Christian Baron, Franz Narberhaus, Erh-Min Lai

**Affiliations:** 1 Institute of Plant and Microbial Biology, Academia Sinica, Taipei, Taiwan; 2 Lehrstuhl für Biologie der Mikroorganismen, Ruhr-Universität Bochum, Bochum, Germany; 3 Biology Department, McMaster University, Hamilton, ON, Canada; 4 Département de Biochimie, Université de Montréal, Montréal, QC, Canada

## Abstract

*Agrobacterium tumefaciens* is a Gram-negative plant-pathogenic bacterium that causes crown gall disease by transferring and integrating its transferred DNA (T-DNA) into the host genome. We characterized the chromosomally encoded alpha-crystallin-type small heat-shock protein (*α*-Hsp) HspL, which was induced by the virulence (*vir*) gene inducer acetosyringone (AS). The transcription of *hspL* but not three other *α*-Hsp genes (*hspC*, *hspAT1*, *hspAT2*) was upregulated by AS. Further expression analysis in various *vir* mutants suggested that AS-induced *hspL* transcription is not directly activated by the VirG response regulator but rather depends on the expression of VirG-activated *virB* genes encoding components of the type IV secretion system (T4SS). Among the 11 *virB* genes encoded by the *virB* operon, HspL protein levels were reduced in strains with deletions of *virB6*, *virB8* or *virB11*. VirB protein accumulation but not *virB* transcription levels were reduced in an *hspL* deletion mutant early after AS induction, implying that HspL may affect the stability of individual VirB proteins or of the T4S complex directly or indirectly. Tumorigenesis efficiency and the VirB/D4-mediated conjugal transfer of an IncQ plasmid RSF1010 derivative between *A. tumefaciens* strains were reduced in the absence of HspL. In conclusion, increased HspL abundance is triggered in response to certain VirB protein(s) and plays a role in optimal VirB protein accumulation, VirB/D4-mediated DNA transfer and tumorigenesis.

## INTRODUCTION

*Agrobacterium tumefaciens* is a soil-borne plant-pathogenic bacterium causing crown gall disease in a wide range of plants through an interkingdom DNA delivery system. *A. tumefaciens* is capable of sensing plant-released wound signal molecules such as sugars and phenolic compounds to activate a signal transduction pathway for infection. This event is regulated by the VirA/VirG two-component system encoded by the tumour-inducing (Ti) plasmid in conjunction with ChvE, a chromosomally encoded periplasmic galactose/glucose-binding protein to activate the expression of virulence (*vir*) genes and operons, including *virA*, *B*, *G*, *C*, *D* and *E* (McCullen & Binns, 2006). The transferred DNA (T-DNA) is then processed, followed by transfer of the T-complex and effector proteins via the Ti plasmid-encoded Vir type IV secretion system (T4SS) from bacteria into the host plant cells (Baron, 2006; Christie *et al.*, 2005).

The Ti Vir T4SS is a transmembrane complex consisting of VirD4 and 11 VirB proteins that also assembles T pili (Christie, 2001; Lai & Kado, 2000). Accumulating biochemical and genetic data suggest a model of an ordered VirB/D4 T4SS assembly pathway (Baron, 2006; Christie *et al.*, 2005; Ward *et al.*, 2002). First, VirB8 initiates T4SS assembly and targets VirB1 to the cell pole, where it may locally lyse the cell wall to facilitate T4SS assembly across the double membranes (Judd *et al.*, 2005; Yuan *et al.*, 2005). VirB6, VirB4, VirB7, VirB8, VirB9 and VirB10 then assemble a core complex, which is followed by recruitment of the subunits important for pilus assembly, including VirB2, VirB3 and VirB5 (Krall *et al.*, 2002). The VirB11 homohexameric ATPase may supply energy for VirB2 polymerization across the periplasm to form the T pilus (Atmakuri *et al.*, 2004; Rashkova *et al.*, 2000). Finally, VirB4 and VirD4 are required for substrate translocation, which may be mechanistically linked to a conformational change of VirB10 (Cascales & Christie, 2004a). The T-DNA/substrate is translocated via four discrete steps of sequential interactions with VirD4, VirB11, VirB6/VirB8 and VirB2/VirB9, as demonstrated by T-DNA immunoprecipitation assay (Cascales & Christie, 2004b). Biochemical approaches have identified subassemblies of VirB proteins constituting the ‘core’ components believed to form the translocation channel and the ‘pilus assembly’ complex comprising pilus components and associated factors (Krall *et al.*, 2002; Yuan *et al.*, 2005). In addition to transporting the T-complex and effector proteins from bacteria into plant cells, the VirB/D4 T4SS can translocate an incompatibility group Q (IncQ) plasmid RSF1010 from *A. tumefaciens* into plant cells (Buchanan-Wollaston *et al.*, 1987) or between agrobacteria (Beijersbergen *et al.*, 1992). Hitherto, little has been known about the contribution(s) of non-VirB proteins to the function of the T4SS; the work presented here suggests a role for the small heat-shock protein HspL.

We have previously used proteomics approaches to identify acetosyringone (AS)-induced proteins and discovered AS induction of HspL (Lai *et al.*, 2006). HspL is an alpha-crystallin-type small heat-shock protein (*α*-Hsp) that contains a characteristic *α*-crystallin domain (Narberhaus, 2002). *α*-Hsps are a diverse protein family of low-molecular-mass chaperones that exist universally in most organisms, including animals, plants, bacteria and archaea. *Rhizobiaceae* contain a large number of *α*-Hsp genes, but little is known about their function except for the heat shock induction and chaperone-like activities of some of them; that is, they prevent model substrates from heat-induced aggregation (Munchbach *et al.*, 1999a, b; Rosen *et al.*, 2002; Studer & Narberhaus, 2000). In *A. tumefaciens*, there are at least four *α*-Hsp genes: *hspC* (*atu0375*) encoded on the circular chromosome, *hspL* (*atu3887*) encoded on the linear chromosome and *hspAT1* (*atu5052*) and *hspAT2* (*atu5449*), both encoded on the pAT plasmid (Balsiger *et al.*, 2004). The latter three are induced by heat shock, and heat induction of *hspL* is regulated by *rpoH,* which encodes an alternative *σ*^32^-like transcription factor (Rosen *et al.*, 2002; Balsiger *et al.*, 2004)

In this study, we characterized the regulation of HspL and its function in the virulence of *A. tumefaciens*. The results indicate that AS-induced HspL protein accumulation is regulated in a VirB*-*dependent manner. Further molecular and functional analyses suggest that HspL protein is required for optimal VirB protein accumulation, which may be important for efficient VirB/D4-mediated DNA transfer and virulence.

## METHODS

### Bacterial strains and growth conditions.

Bacterial strains and plasmids used in this study are listed in Table 1. For *vir* gene induction, *A. tumefaciens* cells grown overnight at 25 °C in 523 broth (Kado & Heskett, 1970) with appropriate antibiotics were harvested by centrifugation (8000 ***g***, 10 min) and resuspended in fresh I-medium (AB-MES, pH 5.5) (Lai & Kado, 1998) without antibiotics, at OD_600_ ∼0.1. After growth at 25 °C to OD_600_ ∼0.2, the cells were further cultured at 25 °C for different times in the presence of 200 μM acetosyringone (AS) (Sigma-Aldrich) (0.1 %, v/v, of 200 mM stock dissolved in DMSO) until harvesting. The controls were grown in the same conditions without any treatment or treated with DMSO, the solvent used to dissolve AS. The concentrations of antibiotics used were: 100 μg ampicillin (Ap) ml^−1^, 20 μg tetracycline (Tc) ml^−1^ and 10 μg gentamicin (Gm) ml^−1^ for *Escherichia coli*; 50 μg erythromycin (Em) ml^−1^, 50 μg rifampicin (Rm) ml^−1^, 250 μg spectinomycin (Sp) ml^−1^, 20 μg Tc ml^−1^ and 50 μg Gm ml^−1^ for *A. tumefaciens.*

### Plasmid construction.

Details of the primers used in this study are given in Supplementary Table S1, available with the online version of this paper. The techniques used for DNA cloning and PCR followed standard protocols (Sambrook & Russell, 2001). Plasmid DNA was isolated using the Plasmid Miniprep Purification kit provided by GeneMark. To construct plasmids for promoter activity assay, DNA fragments of the *hspL*, *hspC*, *hspAT1*, *hspAT2* and *virB* promoter regions were amplified by PCR and digested with *Sp*eI and *Hin*dIII prior to ligation into the promoter-probe vector pRU1156 at the same sites. The translational fusion between the first three amino acids of HspL and GFP (HspL_Δ4–160_-GFP) was constructed by ligating *Hin*dIII/*Spe*I-digested hspLt PCR product and *Spe*I/*Xba*I-digested gfp PCR product into the *Hin*dIII/*Xba*I site of pRU1156. To generate the *hspL* deletion mutant, the plasmids pEML651 and pEML776 were constructed for gene replacement experiments. Plasmid pEML651 was constructed by ligating *Pst*I/*Eco*RI-digested HspL1 PCR product (upstream of *hspL*), *Eco*RI-digested Gm^R^ gene cassette and *Eco*RI/*Sal*I-digested HspL2 PCR product (downstream of *hspL*), into the *Pst*I/*Sal*I sites of the suicide vector pEML649. Plasmid pEML649 was generated by ligating a *Bam*HI-digested *sacB* PCR product into pJM22 at the *Bam*HI site. Plasmid pEML776 was constructed by ligating *Pst*I/*Eco*RI-digested HspL1 PCR product and *Eco*RI/*Sal*I-digested HspL2 PCR product into the *Pst*I/*Sal*I sites of the suicide vector pJQ200KS. Plasmid pHspL was constructed by ligating a *Hin*dIII-digested HspL PCR product (containing promoter and ORF) into the same site of pEML652 for complementation test. To produce His-tagged HspL proteins, the DNA fragment containing the *hspL* ORF without the stop codon was amplified by PCR with specific primers, digested with *Nd*eI and *Xh*oI, and inserted at the same site of pET-22b(+) to result in plasmid pETHspL. The plasmid constructs obtained were confirmed by restriction mapping and DNA sequencing.

### GFP quantification.

To quantify the GFP activities of *A. tumefaciens* cells expressing a *gfp* transcriptional or translational fusion, the bacterial cells were collected and normalized to OD_600_ 0.2 with 0.9 % NaCl. A 100 μl cell suspension was loaded into a Nunc F96 MicroWell plate and analysed for GFP fluorescence with a multilabel plate reader (Chameleon; Hidex Ltd) at 535/485 nm for emission and excitation. Promoter activity was expressed as relative fluorescence units (RFU) after subtracting the fluorescence signal detected from a vector control strain and normalized at OD_600_ 0.1.

### Real-time RT-PCR.

*A. tumefaciens* strain NT1RE(pJK270) was grown for 16 h at 25 °C in I-medium with the addition of DMSO or AS. Total RNA was extracted (Zuber & Losick, 1983) and subjected to reverse transcription with SuperScript III RNase H^−^ Reverse Transcriptase (Invitrogen) (Lai *et al.*, 2006) with the appropriate 3′ primers (Supplementary Table S1). All primers were designed with the software primer express 2.0 (Applied Biosystems). PCR was performed in 25 μl SYBR Master Mix with 100 ng template cDNA and use of an ABI PRISM 7900 HT Sequence Detection System (Applied Biosystems) according to the methods described previously (Lin *et al.*, 2008). To compare data from different PCR runs or cDNA samples, *C*_T_ values for all target genes were normalized to the *C*_T_ value of *16S rRNA*, a constitutively expressed gene with approximately equal PCR efficiency in cells treated with AS or DMSO.

### Gene replacement by double crossover.

Plasmid pEML651 was used to generate the *hspL* deletion mutant with replacement of the Gm^R^ gene cassette, and plasmid pEML776 was used to generate the markerless *hspL* deletion mutant in *A. tumefaciens* strain NT1RE. Plasmid pE1962-Sp was used to generate NT1RE-Sp, the recipient strain for RSF1010 conjugation assay, by transfer of the Sp^R^ gene cassette into the *pgl/picA* locus of *A. tumefaciens* strain NT1RE. A 5 μl volume of overnight culture (grown in LB broth without antibiotics) of *E. coli* strain S-17 containing the respective plasmid and *A. tumefaciens* strain NT1RE were mixed and incubated at 28 °C on LB agar overnight. The bacterial cells were then streaked out on LB agar containing Em, Rm and Km and incubated at 28 °C for 2 days to obtain the first crossover events. Three colonies were randomly selected and streaked out on the same selection medium for further colony purification. Each of three independent colonies was grown in 5 ml I-medium without antibiotics at 20 °C overnight; serial dilutions (up to 10^−2^) were plated onto 523 agar containing 5 % (w/v) sucrose and incubated at 20 °C for 4–7 days. The colonies were then selected for the respective antibiotic resistance and confirmed by colony PCR. The Ti plasmid pJK270 was transferred into the confirmed mutants by conjugation.

### HspL antibody production.

The overexpression of HspL-His followed the instructions of the pET user manual (Novagen, EMD Biosciences). HspL-His was purified with use of Ni-NTA His Bind resins (Novagen), following the manufacturer's instructions. A 1 mg sample of purified HspL-His protein was separated by 15 % (w/v) glycine SDS-PAGE (Sambrook & Russell, 2001), followed by Coomassie brilliant blue R-250 staining (Sambrook & Russell, 2001). The major 19 kDa protein, corresponding to the putative HspL-His, was cut out for polyclonal antibody production in rabbits (GlycoNex, Taipei, Taiwan).

### Western blot analysis.

Proteins were resolved by glycine SDS-PAGE (Sambrook & Russell, 2001) or Tricine SDS-PAGE (Schagger & von Jagow, 1987). Western blot analysis was performed as described previously (Lai & Kado, 1998) with use of primary polyclonal antibodies against HspL, VirB (Baron *et al.*, 2001; Shirasu & Kado, 1993), VirD4 (Chen & Kado, 1996), VirE2 (Baron *et al.*, 2001) and neomycin phosphotransferase II (NptII) (Sigma-Aldrich) followed by secondary antibody using horseradish peroxidase (HRP)-conjugated goat anti-rabbit IgG (chemichem) and detection by the use of the Western Lightning System (Perkin Elmer). Chemiluminescent signals were visualized on X-ray film (Kodak).

### Tumour assay on potato tuber discs.

Quantitative tumorigenesis assays with potato tuber discs were as described previously (Shurvinton & Ream, 1991; Wu *et al.*, 2008) except that bacterial cells at OD_600_ 0.4–0.6 were collected and resuspended in PBS at 10^8^ and 10^7^ c.f.u. ml^−1^ for inoculation. The potato tuber discs were placed on water agar, infected with 10 μl bacterial culture and incubated at 24 °C for 2 days. Discs were then placed on water agar supplemented with 100 μg timentin ml^−1^ to kill bacteria and incubated at 24 °C for 3 weeks before tumours were scored.

### Conjugal transfer analysis of IncQ plasmid RSF1010.

The conjugation assay was as described by Fullner & Nester (1996) with minor modifications. The donor strains were NT1RE(pJK270) and its derivatives and the recipient strain was NT1RE-Sp. Cultures of donor and recipient strains were grown overnight at 25 °C in 523 broth with antibiotics. The cells of donor and recipient strains were collected by centrifugation (8000 ***g***, 5 min) and resuspended in fresh I-medium without antibiotics to OD_600_ ∼0.1. After growth at 25 °C with shaking for 6 h, 200 μM AS was added to the cultures, which continued to grow at 25 °C for an additional 2 h. Donor and recipient cells were mixed together at a ratio of 10 : 1, and 10 μl of the mating mix was spotted on sterilized 1 cm^2^ nylon paper placed on I-medium agar in the presence of 200 μM AS. After incubation at 25 °C for 3 days, the cells from the nylon paper were resuspended in 1 ml 0.9 % NaCl. The bacterial suspensions with or without dilution were plated onto 523 agar supplemented with appropriate antibiotics and incubated at 28 °C for 2 days to select the transconjugants (Em^R^, Gm^R^, Sp^R^), input donors (Em^R^, Gm^R^), and recipients (Em^R^, Sp^R^). The number of transconjugants present in the selected input donors was ignored because of their low frequency (∼10^−5^) in the population.

## RESULTS

### AS-induced *hspL* expression

**Fig. 1. f1:**
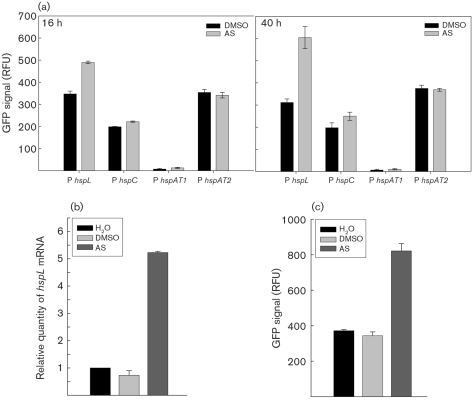
AS-induced *hspL* expression. (a) Relative GFP signal of *A. tumefaciens* strain NT1RE(pJK270) containing a *gfp* transcriptional fusion to the promoter of *hspL*, *hspC*, *hspAT1* or *hspAT2*. The bacteria were grown at 25 °C for 16 or 40 h in I-medium with the addition of DMSO or AS. (b) Quantitative RT-PCR analysis of the *hspL* mRNA level of strain NT1RE(pJK270) and (c) relative GFP signal of strain NT1RE(pJK270) expressing HspL_Δ4–160_-GFP fusion protein (driven by its native promoter), grown at 25 °C for 16 h in I-medium without (H_2_O) or with DMSO or AS. The relative GFP signals for promoter activity are shown as the mean±sd of three independent experiments.

**Fig. 2. f2:**
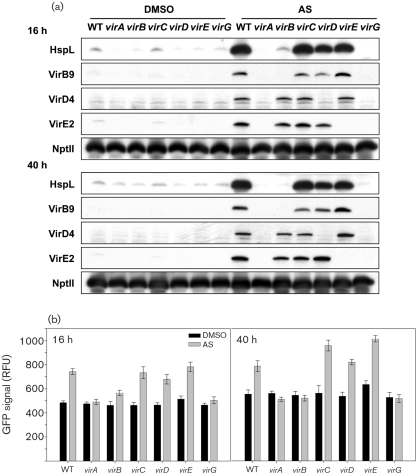
AS-induced HspL protein accumulation is regulated in a VirB-dependent manner. (a) Western blot analysis of HspL, VirB9, VirD4, VirE2 and NptII when wild-type and different *vir* mutants were grown at 25 °C for 16 or 40 h in I-medium with addition of DMSO or AS. (b) Relative GFP signals of *A. tumefaciens* strains containing plasmid pRUhspLp, expressing the P*hspL-gfp* transcriptional fusion, grown at 25 °C for 16 or 40 h in I-medium with DMSO or AS. Strains: WT, NT1RE(pJK270); *virA*, NT1RE(pJK107); *virB*, NT1RE(pJK502); *virC*, NT1RE(pJK702); *virD*, NT1RE(pJK105); *virE*, NT1RE(pJK505); *virG*, NT1RE(pJK710). The relative GFP signals for promoter activity are shown as the mean±sd of three independent experiments. Western blotting was performed for at least three independent experiments with similar results. NptII protein levels were determined as the controls.

The small heat-shock protein HspL was previously identified as an AS-induced protein in *A. tumefaciens* (Lai *et al.*, 2006). To determine whether AS also induces the three other *α*-Hsp genes (*hspC*, *hspAT1*, *hspAT2*), we analysed the promoter activity of each *α*-Hsp gene transcriptionally fused to *gfp*. The P*hspL-gfp* transcriptional fusion was upregulated 1.5- to 2-fold in cells grown in the presence of AS for 16 h or 24 h as compared with the non-induced (DMSO) controls (Fig. 1a[Fig f1]). In contrast, the promoter activities of *hspC*, *hspAT1* and *hspAT2* were not affected by AS treatment. Quantitative RT-PCR, analysis of translational fusion and Western blot analysis were carried out to monitor the individual steps of *hspL* expression. We detected about 5- to 6-fold increased *hspL* mRNA level (Fig. 1b[Fig f1]), 2-fold increase of HspL_Δ4–160_-GFP translational efficiency (Fig. 1c[Fig f1]), and 50-fold higher HspL protein level (Fig. 2a[Fig f2]) in cells grown in the presence of AS for 16 h in comparison to the DMSO or H_2_O controls. These data suggest that AS-induced transcription of *hspL* is a specific response rather than a general effect of AS treatment on heat-shock gene expression.

### HspL protein accumulation is induced by AS in a VirB protein*-*dependent manner

Similar to other known Vir proteins that are regulated by the VirA/VirG two-component system, AS-induced HspL accumulation is also dependent on *virA* and *virG* (Lai *et al.*, 2006). Interestingly, in contrast to the presence of VirG-binding sequences (*vir* box) in the promoters of known *vir* regulon genes (Cho & Winans, 2005), the apparent absence of a *vir* box within the putative *hspL* promoter region suggests that *hspL* is not directly activated by the VirG response regulator. To address this question, the P*hspL-gfp* transcriptional fusion construct was transformed into different *vir* mutants. As expected, *hspL* promoter activity was upregulated by AS in the wild-type and the *virC*, *virD* and *virE* mutants, but not in the *virA* or *virG* mutants, after either 16 h or 40 h AS induction (Fig. 2b[Fig f2]). Surprisingly, *hspL* promoter activity was also compromised in a polar *virB3* mutant (Fig. 2b[Fig f2]) and the steady-state level of AS-induced *hspL* mRNA was also diminished in the *virB3* mutant (data not shown). Furthermore, HspL protein levels were increased by AS in the wild-type and the *virC*, *virD* and *virE* mutants but not in the polar *virB3* mutant (Fig. 2a[Fig f2]). The VirA/VirG two-component system is still functional in the *virB3* polar mutant because other Vir proteins such as VirD4 and VirE2 were still induced by AS (Fig. 2a[Fig f2]). Taken together, these data suggest that AS-induced *hspL* transcription is not directly activated by the response regulator VirG but rather is linked to the expression of *virB* genes.

**Fig. 3. f3:**
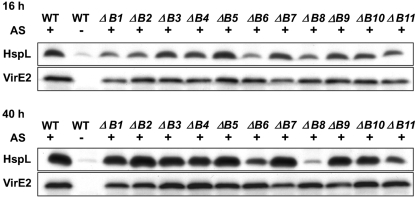
HspL protein accumulation is compromised in the *virB6*, *virB8* and *virB11* nonpolar deletion mutants: Western blot analysis of HspL and VirE2 in *A. tumefaciens* strains containing octopine-type Ti plasmid pTiA6 and its variants. A348, the wild-type strain, was treated with AS (+) or DMSO (−) and each of the *virB* non-polar deletion mutants (Δ*B1*–Δ*B11* represent deletions of *virB1* to *virB11*) induced by AS were determined. Western blotting was performed for at least three independent experiments with similar results. VirE2 protein levels were determined as the controls.

To determine which *virB* gene(s) are responsible for AS-induced HspL protein accumulation, individual *virB* nonpolar deletion mutants were analysed, and the HspL protein was detected by Western blot analysis. Reduced levels of HspL protein were observed in the *virB1* and *virB2* mutants, but only at 16 h after AS induction (Fig. 3). More clearly, after 16 h and 40 h of AS induction, HspL protein levels were reduced in *virB6*, *virB8* and *virB11* deletion strains as compared with the wild-type and the other *virB* mutants. VirE2 levels, as a control, were normal in these strains. These data suggest that HspL protein accumulation is likely induced by the expression of one or a subset of VirB proteins.

### The absence of HspL causes reduced VirB protein accumulation at an early stage of AS induction without affecting *virB* transcription

**Fig. 4. f4:**
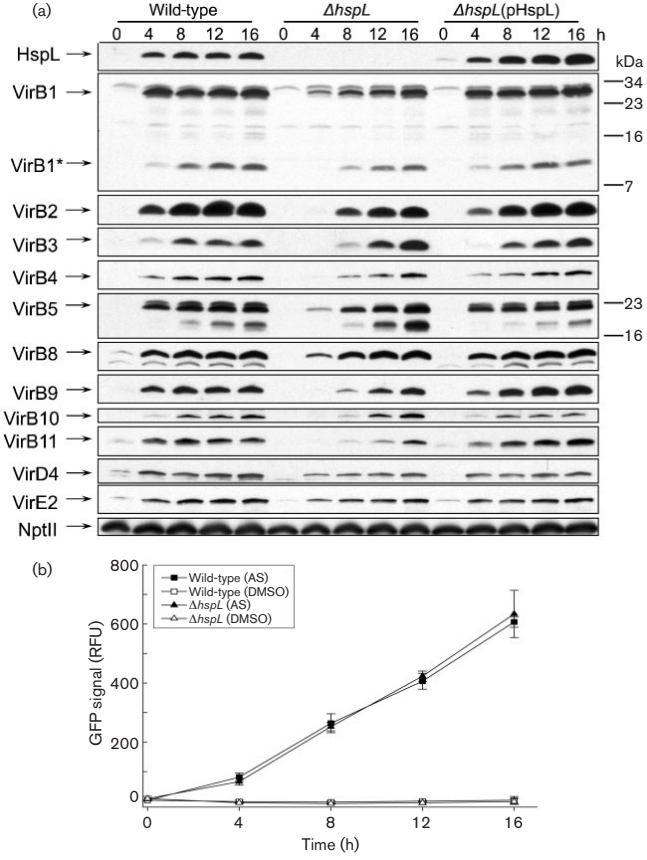
The absence of HspL causes reduced VirB protein accumulation at early stages of AS induction without affecting *virB* operon transcription. (a) Wild-type NT1RE(pJK270), Δ*hspL* (*hspL* deletion mutant, EML770) and Δ*hspL*(pHspL) (complemented strain, EML815) were grown in I-medium at 25 °C in the presence of AS to induce *vir* gene expression. The total cell lysates were subjected to Tricine-SDS-PAGE followed by Western blot analysis. Numbers on the right are molecular masses of reference proteins in kDa. NptII served as an internal control. At least three independent experiments were carried out with similar results. (b) Relative GFP signal of *A. tumefaciens* NT1RE(pJK270) or Δ*hspL* (*hspL* deletion mutant, EML770) containing plasmid pRUvirBp expressing the P*virB-gfp* transcriptional fusion. The bacteria were grown at 25 °C in I-medium with the addition of DMSO or AS and collected at different times for GFP quantification. The relative GFP signals for *hspL* promoter activity are shown as the mean±sd of at least three independent experiments.

We investigated the physiological role of HspL protein expression and accumulation in response to certain VirB proteins. Small heat-shock proteins are generally thought to bind selectively to non-native proteins to prevent their aggregation and degradation (Narberhaus, 2002; Sun & MacRae, 2005). Thus, HspL might play a role as a chaperone in stabilizing VirB proteins and thereby contribute to efficient T-DNA transfer and tumorigenesis. To test this hypothesis, we determined the effect of deletion of *hspL* on VirB protein accumulation. The loss of HspL in the deletion mutant and its complementation by expression of *hspL* (driven by its native promoter) on an IncP plasmid was demonstrated by Western blotting (Fig. 4a[Fig f4]). The level of all VirB proteins analysed was lower in the *hspL* deletion mutant than in the wild-type soon after AS induction (4 h and 8 h). In contrast, levels of other AS-induced proteins such as VirD4 and VirE2 and the internal control protein NptII were not substantially reduced, which suggests that AS-induced HspL acts mainly on VirB proteins. The effects were complemented by *hspL* expression *in trans* (Fig. 4a[Fig f4]). In contrast, *hspL* plays no role in AS-induced *virB* transcription because the *virB* promoter was induced by AS at similar levels in both the wild-type and the *hspL* mutant up to 16 h (Fig. 4b[Fig f4]). Taken together, the data suggest that HspL plays a role in optimal VirB protein accumulation likely via maintaining their stability during the assembly process.

### The absence of HspL causes reduced VirB/D4-mediated DNA transfer and tumorigenesis efficiency

In view of the reduced VirB protein accumulation in the *hspL* deletion mutant, we were curious to determine whether HspL is involved in the VirB/D4-mediated DNA transfer and virulence of *A. tumefaciens*. An IncQ plasmid, RSF1010, can be transferred between strains of *A. tumefaciens* by the Ti plasmid-encoded VirB/D4 machinery in an AS-induced environment (Beijersbergen *et al.*, 1992). We used this alternative functional T4SS assay to determine whether *hspL* contributes to the conjugal transfer using the RSF1010-derived plasmid pML122ΔKm. The transfer efficiency was consistently reduced, by 30 % on average, in the *hspL* mutant as compared with the wild-type (Table 2). The mobilization ability of the *hspL* deletion mutant was restored and even increased by complementation with an *hspL*-expressing plasmid, which indicates that HspL contributes to efficient mobilization of pML122ΔKm between *A. tumefaciens* strains. The observed mobilization of pML122ΔKm was indeed mediated by the Ti VirB/D4 T4SS because no transconjugants were detected in the mutant with deletion of the entire *virB* operon (Table 2) or when the conjugation experiment was carried out in the absence of AS (data not shown). We also determined whether the deletion of *hspL* affects the function of another Ti plasmid-encoded T4SS, the *trb* locus, which mediates the conjugal transfer of the Ti plasmid between agrobacteria (Li *et al.*, 1998; von Bodman *et al.*, 1989). The conjugal transfer efficiency of pTiC58-derived pJK270 was not affected in the *hspL* deletion mutant or in its complemented strain as compared with in the wild-type (data not shown), which suggests that HspL is not a general factor involved in DNA and plasmid transfer. Thus, HspL promotes RSF1010 conjugal transfer, which further substantiates its role in Ti plasmid-encoded VirB/D4 T4SS-dependent function.

**Fig. 5. f5:**
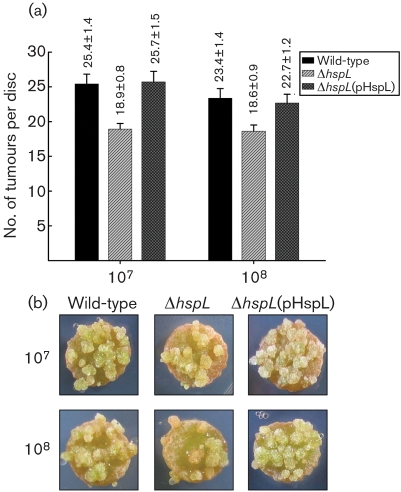
Quantitative tumorigenesis assay on potato tuber discs. (a) Wild-type NT1RE(pJK270), Δ*hspL* (*hspL* deletion mutant, EML770) and Δ*hspL*(pHspL) (complemented strain, EML815) were examined for their tumorigenesis efficiency on potato tuber discs by inoculation at 10^8^ and 10^7^ c.f.u. ml^−1^. Tumorigenesis efficiency is expressed as the number of tumours per disc (mean±se, calculated from results of 60 potato tuber discs for each analysed strain in each independent experiment). (b) Representative results; at least four independent experiments were carried out with similar results.

To determine the effects on *A. tumefaciens* virulence, quantitative tumorigenesis assays on potato tuber discs were used to determine the effect of HspL on tumour formation. The tumorigenesis efficiency was consistently 20–25 % lower in the *hspL* mutant than in the wild-type with inoculation of 10^7^ and 10^8^ c.f.u. ml^−1^ of bacterial cells (Fig. 5). Complementation of *hspL* from a plasmid in the *hspL* deletion mutant restored tumour formation to wild-type levels, which indicated that the attenuated virulence of the *hspL* mutant was caused specifically by the loss of *hspL* (Fig. 5). The reduced tumorigenesis efficiency caused by the deletion of *hspL* was also observed in infected *Arabidopsis thaliana* roots (Supplementary Fig. S1). These data strongly suggest that HspL contributes to promote VirB/D4-mediated DNA transfer and disease development.

## DISCUSSION

Most of the components required for conjugal DNA transfer and tumour formation by *A. tumefaciens* are encoded on the Ti plasmid. In this study, we demonstrated that the chromosomally encoded small heat-shock protein HspL is induced upon expression of certain VirB proteins, the major components of the Ti VirB/D4 T4SS required for gene transfer to plants in *A. tumefaciens*. Our genetic and functional evidence suggests a role for HspL in promoting VirB protein stability, VirB/D4-mediated DNA transfer and tumorigenesis.

The VirB-induced HspL expression resembles the induction of heat-shock proteins and proteases via the extracytoplasmic (or envelope) stress responses observed in many Gram-negative bacteria (Raivio, 2005; Rowley *et al.*, 2006). The known envelope stress response is regulated via the CpxAR two-component regulatory system or the alternative sigma factor *σ*^E^ (Raivio, 2005). In *E. coli*, the expression and assembly of functional plasmid R1-determined T4SS pili and of type IV bundle-forming pili were found to elicit the envelope stress responses via the CpxAR two-component regulatory system (Nevesinjac & Raivio, 2005; Zahrl *et al.*, 2006). The discovery of VirB-induced HspL in this study and the identification of Cpx regulation of *α*-Hsp genes *ibpA* and *ibpB* in *E. coli* (Lau-Wong *et al.*, 2008) suggest the involvement of *α*-Hsps in envelope stress responses. To our knowledge, however, HspL is the first *α*-Hsp demonstrated to be involved in the function of a protein secretion system. The mechanism of *hspL* induction is not clear because CpxAR components could not be identified in the *A. tumefaciens* C58 genome based on blast analysis. RpoH (*σ*^32^) is required for heat-shock-induced HspL protein accumulation (Rosen *et al.*, 2002) and likely regulated at the transcriptional level due to the presence of an RpoH-dependent promoter of *hspL* (Balsiger *et al.*, 2004). However, RpoH was not essential for AS-induced HspL protein accumulation under non-heat-shock conditions because the HspL protein level accumulated to the wild-type level in the *A. tumefaciens rpoH* mutant after AS induction at 25 °C (data not shown). Therefore, the expression of certain VirB proteins may trigger an as yet unknown regulator(s) that upregulate(s) *hspL* expression in *A. tumefaciens* under non-heat-shock conditions.

Although the assembly of pili by the IncFII plasmid R1 T4SS triggered the envelope stress response and a decreased response was observed in a *traA* pilin mutant (Zahrl *et al.*, 2006), the exact T4SS component that mediates its induction is unknown. Our data indicate that AS-induced HspL protein accumulates to the wild-type level in most of the single *virB* deletion mutants after 40 h induction (Fig. 3), which suggests that T4SS-induced HspL protein accumulation requires neither the presence of an intact secretion system nor the formation of the T pilus. The evidence that the HspL protein level was markedly reduced in the *virB6*, *virB8* and *virB11* deletion mutants suggests that HspL protein accumulation may be triggered by one or a subset of T4SS components. We noticed that VirB8 and VirB11 protein levels were reduced in the *virB6* deletion mutant (data not shown). Since deletion of *virB6* had a negative effect on downstream gene expression (*virB7–virB11)* (Liu & Binns, 2003), it remains to be determined whether the requirement of VirB6 in triggering HspL protein accumulation is direct or indirect. VirB6, VirB8 and VirB11 are inner-membrane components directly involved in the T-DNA/substrate translocation pathway (Cascales & Christie, 2004b); however, T-DNA translocation through this T4SS channel is not required for HspL induction because the VirD4 coupling protein is dispensable for this effect (Fig. 2a, b[Fig f2]). Interestingly, the deletion of *virB8* caused the greatest decrease in HspL protein level (Fig. 3). Because VirB8 is an assembly factor that may initiate T4SS assembly (Baron, 2006), we speculate that *hspL* transcription and its protein accumulation may be triggered by the formation of the early subassembly complex.

Although the VirB protein level was clearly reduced, we did not observe effects on *virB* transcription in the *hspL* deletion mutant as compared with the wild-type (Fig. 4), which suggests that HspL may function as a VirB chaperone. Interestingly, although *hspL* seems to be expressed at a basal level and is upregulated only about twofold by AS at the transcriptional level, based on our promoter activity assay (Fig. 1a[Fig f1]) and microarray data (Anand *et al.*, 2008), HspL protein is barely detectable in the absence of AS but accumulates markedly – about 50-fold – upon AS induction, in a VirB protein-dependent manner (Fig. 2a[Fig f2]). The twofold upregulation of the HspL_Δ4–160_-GFP translational fusion by AS (Fig. 1c[Fig f1]) further suggests a post-translational regulation of AS-induced HspL accumulation. Both a chaperone and its interacting substrate become stabilized when they interact with each other (Narberhaus, 2002; Sun & MacRae, 2005). Thus, we speculate that HspL protein might be stabilized when interacting with its substrates such as VirB proteins and may be rapidly degraded in the absence of its substrate. Likewise, the substrates may be more susceptible to proteolysis in the absence of the chaperone. We are currently investigating whether HspL interacts directly with VirB protein(s) and functions as a VirB chaperone to prevent VirB from aggregation and degradation, thereby maintaining the stability and/or functionality of the individual VirB proteins and/or the assembled T4SS complexes.

The discovery of HspL as a non-VirB factor contributing to T4SS protein stability is novel, but most importantly, the decreased VirB protein accumulation in the absence of HspL also correlates with the reduced tumorigenesis efficiency of the *hspL* mutant as compared with the wild-type (Fig. 5, Supplementary Fig. S1). Obviously HspL plays a specific role for the Ti VirB/D4 T4SS because VirB/D4-mediated RSF1010 transfer but not Trb-mediated Ti plasmid transfer between agrobacteria was decreased in the absence of HspL (Table 2). This specificity was further supported by evidence that *hspL* but none of the other three *α*-Hsp genes (*hspC*, *hspAT1* and *hspAT2*) was upregulated by AS (Fig. 1a[Fig f1]) and no deleterious effects on growth or membrane lipid composition were detected in the absence of *hspL* (data not shown). However, one may argue that HspL does not contribute an essential function for *A. tumefaciens* virulence because the reduced VirB protein accumulation and tumorigenesis efficiency was not as drastic in the *hspL* mutant as the wild-type (Fig. 4a[Fig f4]; compare with Fig. 5, Supplementary Fig. S1). Functional redundancy of *α*-Hsps was found in *E. coli*, in which the simultaneous presence of *α*-Hsps IbpA and IbpB enhanced the stabilization of thermally aggregated proteins as compared with the presence of IbpA or IbpB alone (Matuszewska *et al.*, 2005). Thus, it is possible that the basal-level expression of the other three *α*-Hsps may partially substitute for the function of HspL in VirB protein stability and T4SS function in the absence of HspL. Examining the effect on the stability of VirB proteins/complexes, VirB/D4-mediated DNA transfer and tumorigenesis in single or multiple *α*-Hsp mutants would be an interesting future study.

In general, the expression of bacterial *α*-Hsp genes is low or undetectable under normal growth conditions but is induced to high levels under heat shock or other stress conditions (Narberhaus, 2002). The induction of *α*-Hsp genes was previously reported during bacterial infection with the human pathogens *Mycobacterium tuberculosis* and *Mycobacterium leprae*. The *α*-Hsp genes *acr1* and *acr2* are induced in *M. tuberculosis*-infected macrophages and *acr2* was further demonstrated to be required for the pathogenicity (Stewart *et al.*, 2005, 2006; Wilkinson *et al.*, 2005). The expression of another *α*-Hsp gene, *shsp18*, encoding a surface-exposed antigen of *M. leprae* (Ilangumaran *et al.*, 1994; Lini *et al.*, 2008), was activated in macrophages (Dellagostin *et al.*, 1995). Our findings that the phytopathogen *A. tumefaciens* exploits the VirB-induced HspL expression to promote its tumorigenesis add to the list of *α*-Hsp participation in bacterial virulence. Further study could explore the importance of small heat-shock proteins in the virulence of other bacterial pathogens and elucidate the molecular mechanisms underlying their regulation and involvement in their infection processes under non-heat-shock conditions.

## Figures and Tables

**Table 1. t1:** Bacterial strains and plasmids

**Strain/plasmid**	**Relevant characteristics**	**Reference/source**
***A. tumefaciens***		
A136	Rm^R^, strain C58 cured of the pTiC58 plasmid	Watson *et al.* (1975)
A348	Rm^R^, A136 containing octopine-type Ti plasmid pTiA6	Garfinkel *et al.* (1981)
PC1001–PC1011	Rm^R^, A348 derivatives each containing a *virB* gene deletion from pTiA6	Berger & Christie (1994)
NT1RE	Rm^R^ Em^R^, C58 cured of its pTiC58	Watson *et al.* (1975)
NT1RE-Sp	Rm^R^ Em^R^ Sp^R^, NT1RE containing spectinomycin-resistant gene (*aadA*)	This study
NT1RE(pJK270)	Rm^R^ Em^R^ Km^R^/Nm^R^; pJK270 is pTiC58Tra^C^ with *Tn*5 insertion in the T-DNA region without affecting virulence	Kao *et al.* (1982)
NT1RE(pJK107)	Rm^R^ Em^R^ Km^R^/Nm^R^, *virA* : : *Tn*5 in pTiC58Tra^C^, *virG* mutant	Kao *et al.* (1982)
NT1RE(pJK105)	Rm^R^ Em^R^ Km^R^/Nm^R^, *virD1* : : *Tn*5 in pTiC58Tra^C^, *virD1* polar mutant	Rogowsky *et al.* (1987)
NT1RE(pJK502)	Rm^R^ Em^R^ Km^R^/Nm^R^, *virB3* : : Tn5 in pTiC58Tra^C^, *virB3* polar mutant	Lundquist *et al.* (1984)
NT1RE(pJK505)	Rm^R^ Em^R^ Km^R^/Nm^R^, *virE* : : Tn5 in pTiC58Tra^C^, *virE* polar mutant	Rogowsky *et al.* (1987)
NT1RE(pJK702)	Rm^R^ Em^R^ Km^R^/Nm^R^, *virC1* : : *nptII* in pTiC58Tra^C^, *virC1* mutant	Rogowsky *et al.* (1987)
NT1RE(pJK710)	Rm^R^ Em^R^ Km^R^/Nm^R^, *virG* : : *nptII* in pTiC58Tra^C^, *virG* mutant	Kao *et al.* (1982)
NT1RE(pEL1000)	Rm^R^ Em^R^ Km^R^/Nm^R^, *virB* operon deletion in pJK270	This study
EML770	Rm^R^ Em^R^ Km^R^/Nm^R^ Gm^R^, *hspL* replaced by Gm cassette to generate *hspL* deletion mutant in NT1RE(pJK270)	This study
EML815	Rm^R^ Em^R^ Km^R^/Nm^R^ Gm^R^ Tc^R^, pHspL in EML770 for complementation experiment	This study
EML1057	Rm^R^ Em^R^ Km^R^/Nm^R^, markerless *hspL* deletion mutant in NT1RE(pJK270)	This study
EML1280	Rm^R^ Em^R^ Km^R^/Nm^R^ Tc^R^, pHspL in EML1057 for complementation experiment	This study
***E. coli***		
DH10B	Host for DNA cloning	Invitrogen
S-17	Host for conjugation	Simon *et al.* (1983)
**Plasmids**		
pGEMT-Easy	Ap^R^, TA cloning vector	Promega
pJQ200KS	Gm^R^, plasmid containing Gm^R^ and *sacB* gene for selection of double crossover	Quandt & Hynes (1993)
pUCG Ω1	Gm^R^, broad-host-range Gm^R^ cassettes for site-specific insertion	Schweizer (1993)
pGEMT-Sp	Ap^R^ Sp^R^, pGEM-T-easy vector containing spectinomycin-resistance gene (*aadA*) cassette	Wu *et al.* (2008)
pET-22b(+)	Ap^R^, an *E. coli* overexpression vector to generate C-terminal His-tagged protein	Novagen
pRU1064	Ap^R^ Tc^R^, stable broad-host-range promoter-probe vector containing *gfpUV* and *gusA*	Karunakaran *et al.* (2005)
pRU1156	Ap^R^ Tc^R^, stable broad-host-range promoter-probe vector containing *gfpmut3.1* and *gusA*	Karunakaran *et al.* (2005)
pRUhspLp	Ap^R^ Tc^R^, expression of P*hspL-gfp* transcriptional fusion on pRU1156	This study
pRUhspCp	Ap^R^ Tc^R^, expression of P*hspC-gfp* transcriptional fusion on pRU1156	This study
pRUhspAT1p	Ap^R^ Tc^R^, expression of P*hspAT1-gfp* transcriptional fusion on pRU1156	This study
pRUhspAT2p	Ap^R^ Tc^R^, expression of P*hspAT2-gfp* transcriptional fusion on pRU1156	This study
pRUvirBp	Ap^R^ Tc^R^, expression of P*virB-gfp* transcriptional fusion on pRU1156	This study
pRUhspLt	Ap^R^ Tc^R^, expression of P*hspL-gfp* translational fusion protein HspL_Δ4–160_-GFP on pRU1156	This study
pJM22	Km^R^, vector for M2(Flag) epitope tagging	Janine Maddock
pEML649	Km^R^, pJM22 containing *sacB* gene	This study
pEML651	Km^R^ Gm^R^, up- and downstream fragments of *hspL* and G Ω cassette inserted at *Pst*I/*Sal*I site of pEML649	This study
pEML652	Ap^R^ Tc^R^, pRU1064 digested with *Pst*I to remove reporter gene (*gfpUV* and *gusA*)	Liu *et al.* (2008)
pEML776	Gm^R^, up- and downstream fragments of *hspL* inserted at *Pst*I/*Sal*I site of pJQ200KS	This study
pHspL	Ap^R^ Tc^R^, expression of *hspL* gene containing its promoter and ORF on pEML652	This study
pETHspL	Ap^R^, overexpression of HspL-His in *E. coli*	This study
pML122ΔKm	IncQ plasmid RSF1010 derivative pML122 with removal of *nptII* gene	Labes *et al.* (1990)</xref>
pE1962	Tc^R^, plasmid for introducing gene into the *pgl/picA* locus in the chromosome of *A. tumefaciens*	Lee *et al.* (2001)
pE1962-Sp	Tc^R^ Sp^R^, pE1962 containing Sp^R^ gene cassette	This study

**Table 2. t2:** Effect of *hspL* on mobilization of pML122ΔKm in *A. tumefaciens*

**Donor strain**	**Relevant genotype**	Conjugation frequency (%)*		
		**Experiment 1**	**Experiment 2**	**Experiment 3**
NT1RE(pEL1000)	*virB* operon deletion mutant	<4.35×10^−8^ (<0.27)	<3.61×10^−8^ (<0.29)	<4.14×10^−8^(<0.21)
NT1RE(pJK270)	Wild-type	1.63×10^−5^ (100)	1.26×10^−5^ (100)	1.96×10^−5^ (100)
EML1057	Δ*hspL*	1.08×10^−5^ (66.02)	7.96×10^−6^ (63.2)	1.56×10^−5^ (79.3)
EML1280	Δ*hspL*(pHspL)	2.98×10^−5^ (182.25)	1.64×10^−5^ (130.2)	4.01×10^−5^ (204.0)

*The conjugation frequency is expressed as number of transconjugants per input donor.

## Abbreviations

- AS, acetosyringone
- *α*-Hsp, *α*-crystallin-type small heat-shock protein
- RFU, relative fluorescence units
- T4SS, type IV secretion system
- T-DNA, transferred DNA
- Ti, tumour-inducing (plasmid)
